# Follow-Up Programs for Childhood Cancer Survivors in Europe: A Questionnaire Survey

**DOI:** 10.1371/journal.pone.0053201

**Published:** 2012-12-31

**Authors:** Stefan Essig, Roderick Skinner, Nicolas X. von der Weid, Claudia E. Kuehni, Gisela Michel

**Affiliations:** 1 Institute of Social and Preventive Medicine (ISPM), University of Bern, Bern, Switzerland; 2 Department of Pediatric and Adolescent Hematology/Oncology, and Children’s BMT Unit, Great North Children’s Hospital, Newcastle upon Tyne, United Kingdom; 3 Pediatric Oncology/Hematology, University Children's Hospital Basel (UKBB), Basel, Switzerland; Academic Medical Center, Netherlands

## Abstract

**Background:**

For many childhood cancer survivors follow-up care is important long after treatment completion. We aimed to describe the availability and characteristics of long-term follow-up programs (LTFU) across Europe, their content and aims, their problems, and to assess opinions on different models of LTFU.

**Methodology/Principal Findings:**

We asked 179 pediatric oncology institutions in 20 European countries to complete an online survey on LTFU available at their institution. Of 110 respondents (62% response), 66% reported having LTFU for pediatric survivors, 38% for adult survivors of childhood cancer. Availability varied widely across European regions, from 9% of institutions in Northern Europe reporting LTFU for adult survivors to 83% of institution on the British Isles reporting LTFU for pediatric survivors. Pediatric and adult LTFU were usually located in pediatric hospitals and run by pediatric oncologists. Content of follow-up included screening for adverse outcomes and health education. Important problems included lack of time, personnel and funding. Most institutions without LTFU reported that they would like to offer a program (86%).

**Conclusion/Significance:**

Despite general agreement on the need of follow-up care, there is still a lack of well-organized LTFU for survivors of childhood cancer across Europe.

## Introduction

Specialists promote long-term follow-up programs for childhood cancer survivors including comprehensive, evidence-based health care and education (LTFU). Relevant guidelines have been published [1]–[5] and the National Cancer Survivorship Initiative (NCSI) recently recommended that new models of follow-up care should be implemented [6], [7]. All these efforts address the problem of cancer- and treatment-induced late adverse outcomes. Around two-thirds of childhood survivors develop late effects, even long after treatment is completed, including a wide range of physical and psychosocial problems, such as endocrine or cardiac problems, second malignancies, psychological distress, educational problems or increased late mortality [8]–[12].

There is little data on the availability of LTFU for childhood cancer survivors in Europe. In other regions, surveys indicated that only some survivors regularly attend well-organized LTFU. In a recent survey among institutions of the Children’s Oncology Group (COG), located predominantly in the USA, 59% of respondents reported that LTFU is available to pediatric survivors, and 47% to adult survivors [13]. In a Canadian survey, the respective proportions were 87% and 53% [14].

To describe the availability of LTFU for pediatric and adult survivors of childhood cancer, we performed a questionnaire survey among European institutions treating children with cancer. Our aims were to: 1) describe the availability and characteristics of LTFU for survivors across Europe; 2) describe the content and aims; 3) and problems of LTFU; and 4) assess opinions on optimal LTFU.

## Methods

### Ethics Statement

All respondents agreed to take part in this study. According to local and international guidelines on ethics considerations in research involving human participants, this survey among physicians on health care system issues does not raise any ethical concerns [15], [16]. Therefore, formal ethics approval from an ethical committee was deemed unnecessary.

### Sample/Procedure

We aimed to address all institutions treating patients with childhood cancer across Europe. Because of a recent survey on follow-up care in France [17] and a planned study in Germany, we did not contact the institutions in these two countries. Countries were grouped according to the United Nations definition of European regions, except that we grouped the UK and Ireland into the “British Isles” [18]. This allowed to better differentiate between health systems.

Through web-search and contact with national representatives of the pan-European PanCare late effects network (www.pancare.eu) we compiled a list of all institutions in 20 European countries where children with cancer are treated. We developed an online questionnaire and sent an e-mail inviting heads of the centers to complete the password-protected online questionnaire. If they felt they were not the best person to complete it, we asked center heads to forward the e-mail to the person responsible for follow-up care at their institution. If we did not receive the completed questionnaire within two weeks, we sent an e-mail reminder. If there was still no response, we asked national PanCare representatives to prompt the center head to respond.

### Questionnaire

We defined long-term follow-up programs (LTFU) as: “A model of specialized care dedicated to follow-up of childhood cancer survivors. Specialized care may contain comprehensive, evidence-based health care and education for survivors of childhood cancer. Follow-up only done for participants of clinical trials during trial follow-up should not be included in this definition”.

The questionnaire comprised five sections: 1) information on the respondents/institution; 2) follow-up available at institution; 3) LTFU for survivors in pediatric care (<16–20 years of age); 4) LTFU for adult survivors (age >16–20 years); and, 5) guidelines used for follow-up.

### (Questionnaire S1)

Our first aim was to assess the availability and describe the characteristics of LTFU for pediatric and adult survivors across Europe. To address it, we asked respondents if a follow-up program/clinic was available to pediatric or adult survivors of childhood cancer, respectively. We also asked all respondents about their professional background and the characteristics of their institution and LTFU (frequency and location of program/clinic, staff and survivors involved).

Our second aim, to assess content and aims of follow-up, was addressed by asking if those running the LTFU clinic used relevant guidelines, and if they screened for certain problems and/or educated survivors with specific information.

For our third aim, to describe problems of the LTFU, we provided a list of possible problems and asked respondents to tick the problems they encountered in their LTFU.

Our fourth aim, assessing different models of follow up, was addressed by asking which staff should ideally be involved in follow-up care. We also asked respondents to rank different models for organizing follow-up care, adapted from Wallace and colleagues [19], according to how optimal respondents considered them to be (1 = least, 6 = most optimal).

### Analyses

Analyses were performed with Stata 12.0 (Stata Corporation, Austin, Texas). We used descriptive analyses with chi^2^-tests for most outcomes. We used t-tests to compare the mean of the participants’ estimation of the proportion of survivors attending follow-up at different time periods after treatment. We used separate linear regressions to compare the age range of survivors involved and staff that should ideally be involved in follow-up care (dependent variables) between European regions (independent variable). To compare ratings of optimal models of follow-up care we used Hotelling's T-square statistic to test for the overall differences between means of all models of care and Student’s t-test for post-hoc analyses to tests for differences between two specific models.

## Results

### Respondents

We contacted 179 institutions in 20 European countries. Of these, 110 (62%) completed at least section one of the questionnaire (**Table 1**). Most respondents were pediatric oncologists (n = 82, 88%; the percentage given always reflects the respective proportion of institutions out of those institutions responding to the specific question), worked in a university hospital’s pediatric oncology department (n = 83, 89%), and were involved both in acute care (n = 92, 99%) and follow-up care (n = 91, 98%). These results did not differ significantly between institutions with or without LTFU for pediatric or adult survivors (**Table 2**).

**Table 1:** Institutions contacted and responding, and programs available.

Region	Country	Total number of institutions contacted	Responding institutions		Institutions with LTFU			
					for pediatric survivors^a^		for adult survivors^a^	
		n	n	%	n	%	n	%
**British Isles**		**21**	**15**	**71**	**10**	**83**	**8**	**67**
	Ireland	1	1	100	1	100	0	0
	UK	20	14	70	9	82	8	73
**Northern Europe**		**16**	**11**	**69**	**7**	**64**	**1**	**9**
	Denmark	2	1	50	1	100	0	0
	Finland	5	3	60	3	100	0	0
	Lithuania	1	1	100	0	0	0	0
	Norway	3	1	33	0	0	0	0
	Sweden	5	5	100	3	60	1	20
**Southern Europe**		**83**	**47**	**57**	**22**	**56**	**17**	**45**
	Greece	4	4	100	3	100	2	67
	Italy	54	25	46	12	60	12	63
	Portugal	1	0	0	na		na	
	Slovenia	1	1	100	1	100	1	100
	Spain	23	17	74	6	40	2	13
**Western Europe**		**30**	**20**	**67**	**13**	**72**	**7**	**39**
	Austria	5	3	60	1	50	1	50
	Belgium	8	4	50	3	75	1	25
	Netherlands	8	4	50	4	100	4	100
	Switzerland	9	9	100	5	63	1	13
**Eastern Europe**		**29**	**17**	**59**	**9**	**69**	**2**	**17**
	Czech Republic	2	2	100	1	100	1	100
	Hungary	8	2	25	2	100	0	0
	Poland	16	11	69	5	63	1	14
	Slovak Republic	3	2	67	1	50	0	0
**Total**		**179**	**110**	**61**	**61**	**66 (CI:55–75)**	**35**	**38 (CI: 28–49)**

Abbreviations: LTFU, long-term follow-up program; na, not applicable; CI, 95% Confidence Interval.

a some respondents to the questionnaire did not answer the question on LTFU (available respondents for pediatric programs: N = 93; for adult programs: N = 91).

**Table 2:** Characteristics of responding institutions.

	Institution without LTFU		Institution with LTFU		
	N	%	N	%	p
Total	30	100	63	100	
**Professional background**					
Pediatric oncologist/haematologist	28	93	54	86	0.288
Other	2	7	9	14	
**Type of institution (several possible)**					
Pediatric Oncology/Haematology, University Hospital	29	97	54	86	0.265
Pediatric Oncology/Haematology, Other Hospital	1	3	7	11	
Adult Ward, University Hospital	0	0	2	3	
**In which areas of care are you involved (several possible)**					
Acute care of newly diagnosed patients	30	100	62	98	0.488
Short term FU (<5 years after diagnosis)	30	100	61	97	0.324
Long term FU (>5 years after diagnosis)	28	93	59	94	0.954
Other areas*	4	13	10	16	0.749

Abbreviations: LTFU, long-term follow-up programmes; *other areas: e.g. palliative care, stem cell transplantation, transition.

### Availability and Characteristics

Almost all respondents (n = 90, 97%) indicated that there is some form of follow-up available for survivors. In total, 63 (68%) institutions reported having LTFU available; 66% (n = 61) for pediatric survivors, and 38% (n = 35) for adult survivors. Proportions of institutions providing LTFU varied considerably between European countries and regions (**Table 1**). For pediatric survivors LTFU was available in all regions, with proportions between 56% (Southern Europe) and 83% (British Isles). For adult survivors the availability varied considerably, with proportions between 9% (Northern Europe) and 67% (British Isles). Among 29 respondents who had LTFU for neither pediatric nor adult survivors, 25 (86%) stated that they would like a LTFU at their institution.

#### Frequency and location

LTFU clinics were offered weekly in most institutions (for pediatric survivors: n = 25, 49%; for adult survivors: n = 11, 39%), more frequently in 14 (28%) and 7 (25%) institutions, respectively, and less frequently in 7 (14%) and 6 (21%) institutions, respectively. Most institutions sent survivors an appointment date for their follow-up (LTFU for pediatric survivors: n = 52, 90%; LTFU for adult survivors: n = 24, 75%); others sent an invitation to contact the clinic for an appointment (n = 7, 12%; n = 7, 22%) or left it to the survivor to request an appointment (n = 1, 2%; n = 1, 3%; multiple responses were permitted). Most LTFU clinics for pediatric survivors were situated in a pediatric hospital (n = 56, 98%); 18 LTFU clinics for adult survivors (56%) were situated in a pediatric hospital, and 14 in an adult hospital (44%).

#### Staff involved

LTFU for pediatric survivors was most often run by a pediatric oncologist (n = 36, 64%; in 5 additional institutions [9%] the program was run jointly by a pediatric and medical oncologist). One program was run by a medical oncologist (2%), one by a nurse (2%) and 13 by various combinations of specialists (23%). LTFU for adult survivors was also most often run by a pediatric oncologist (n = 13, 46%; in 1 additional institution [4%] the program was run jointly by a pediatric and medical oncologist). Six programs were run by a medical oncologist (21%), one by a nurse (4%) and 5 by various combinations of specialists (18%).

**Figure 1:**
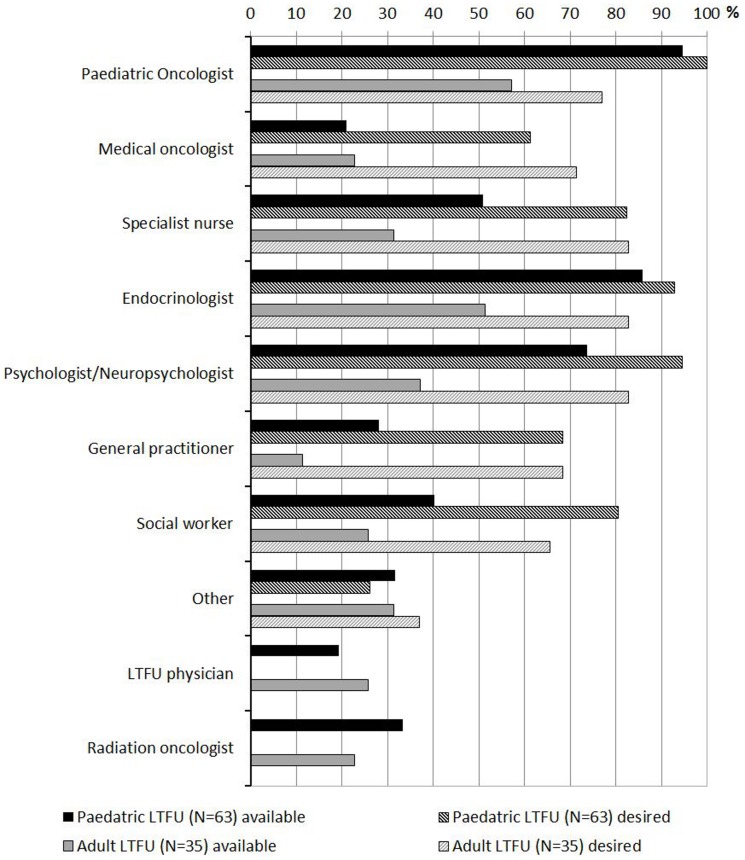
Staff involved in LTFU and staff desired in an optimal model of follow-up care. LTFU: long-term follow-up program.

In most cases, the pediatric oncologist who treated the patient originally was involved in LTFU, both for pediatric and adult survivors (n = 47, 81%; n = 20, 63%). Other staff were involved in all but one LTFU (98%, **Figure 1**).

#### Survivors attending

**Figure 2:**
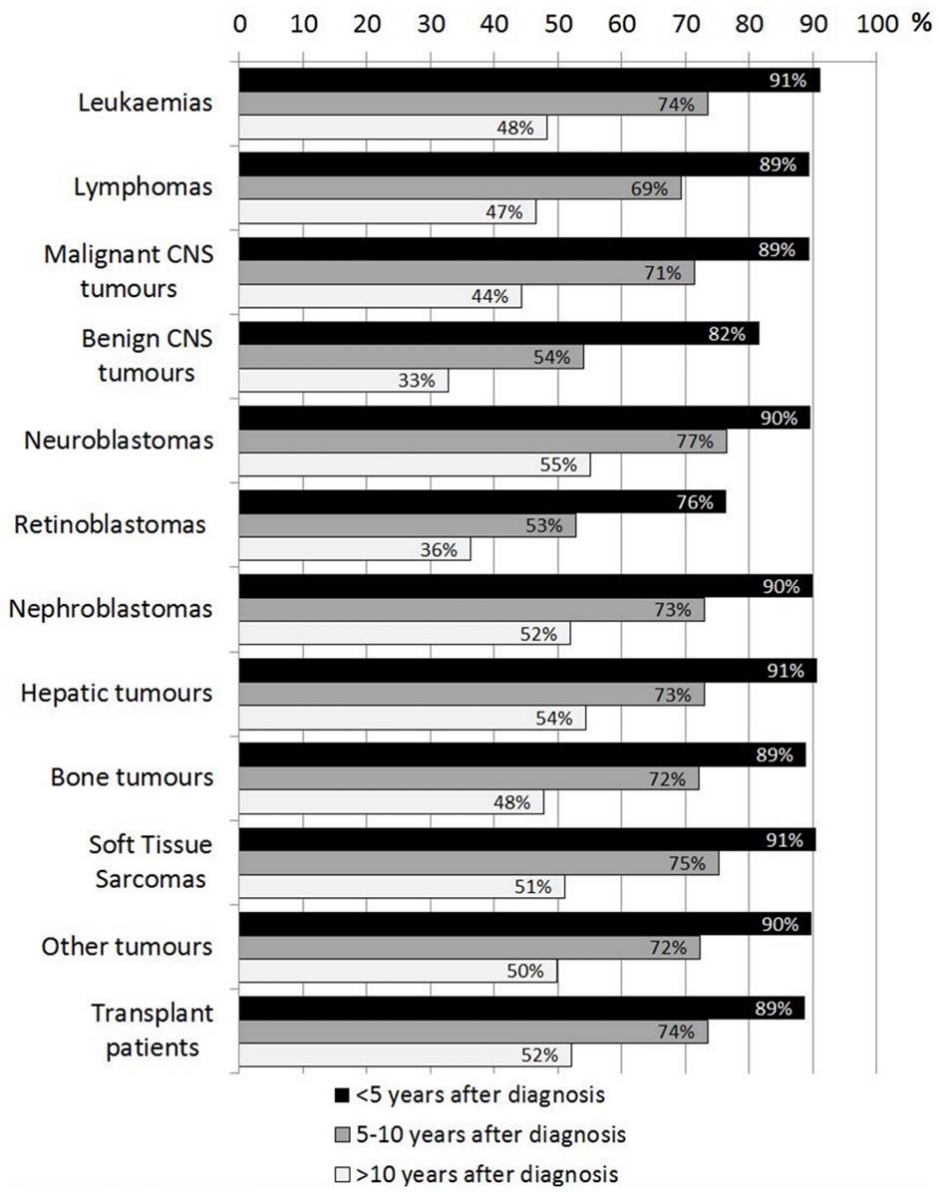
Survivors involved in LTFU: Estimated proportion of pediatric survivors attending follow-up<5, 5–10 and >10 years after diagnosis. LTFU: long-term follow-up program; CNS: central nervous system.

Throughout the first 5 years after diagnosis, respondents estimated that almost all survivors attended LTFU (mean = 88%, SD = 4.4), but the number decreased to 70% over the next 5–10 years (SD = 7.8) and to 48% >10 years after diagnosis (SD = 6.9, all p≤0.001; **Figure 2**). Although intended primarily for pediatric survivors, many institutions included older patients in their follow-up program and the upper age limit varied considerably (mean = 27.1, SD = 15.4, range: 14 to “no age limit”), even within countries (e.g. UK: mean = 30.1, SD = 14.7, range: 18–60; Italy: mean = 28.1, SD = 8.5, range: 16–40; Spain: mean = 22.2, SD = 8.3, range: 14–38). However, there was no significant difference between European regions (F(4,51) = 0.37, p = 0.827).

Eventually, 70% of institutions (n = 39) discharged pediatric survivors, mostly to GPs (n = 24, 42%), adult oncologists (n = 21, 37%) or a transition program (n = 10, 18%; multiple responses were permitted). Among the reasons for discharge were that the patient had reached a certain age (n = 28, 68%), that patients did not want to continue LTFU (n = 11, 27%), lack of resources (n = 3, 7%), and the increasing number of survivors (n = 2, 5%).

Adult survivors started attending adult LTFU from a median age of 18 years (SD = 4.6, range: 16–40). Survivors were eventually discharged in 45% of programs (n = 14), mostly to GPs (n = 9, 28%) or adult oncologists (n = 6, 19%; several responses possible). Among the reasons for discharge were that patients were considered “low risk” for late effects (n = 8; 44%), patients did not want to continue LTFU (n = 6, 33%), other specialists were more appropriate (n = 5, 28%), patients had reached a certain age (n = 4, 22%), and attending a pediatric ward was an obstacle (n = 2, 11%).

### Content of LTFU

**Figure 3:**
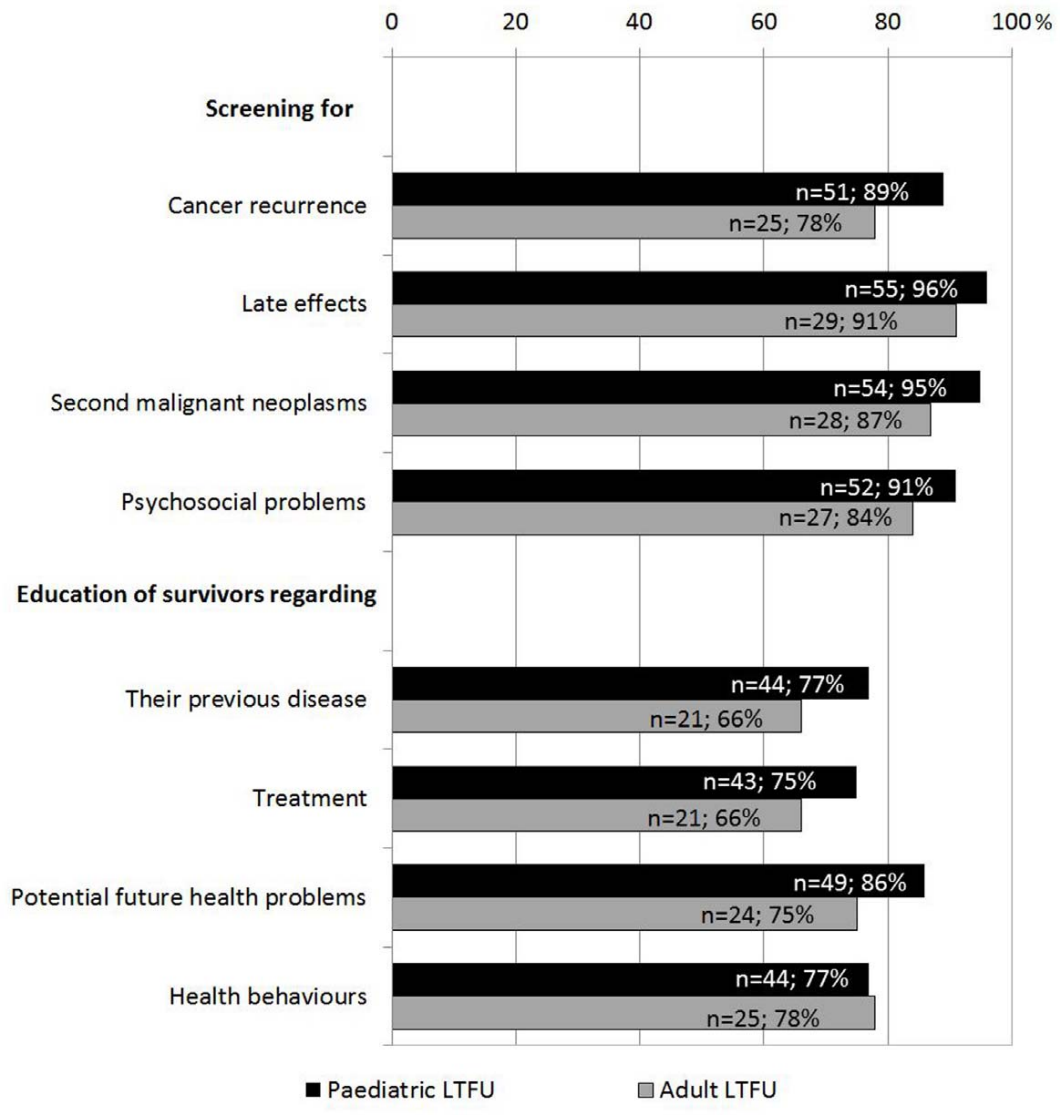
Content of pediatric and adult LTFU. LTFU: long-term follow-up program.

Most respondents reported that they used guidelines to follow both pediatric and adult survivors (n = 48, 89%; n = 25, 81%). Institutions screened for cancer recurrence (**Figure 3**), late effects, second malignant neoplasms, and psychosocial problems. They also educated survivors about their previous disease and treatment, potential future health problems and future health behaviors. There was no difference between European regions (all p>0.05). Detailed results are presented in **Table S1**. A written summary of the cancer treatment was always provided to survivors by 60% (n = 36, pediatric LTFU) and 44% (n = 15, adult LTFU) of institutions, respectively. Many gave general information about late effects to all survivors (n = 34, 64%; n = 21, 68%), while most provided patient-specific information to all survivors (n = 51, 85%; n = 29, 85%).

### Problems Encountered in LTFU

**Figure 4:**
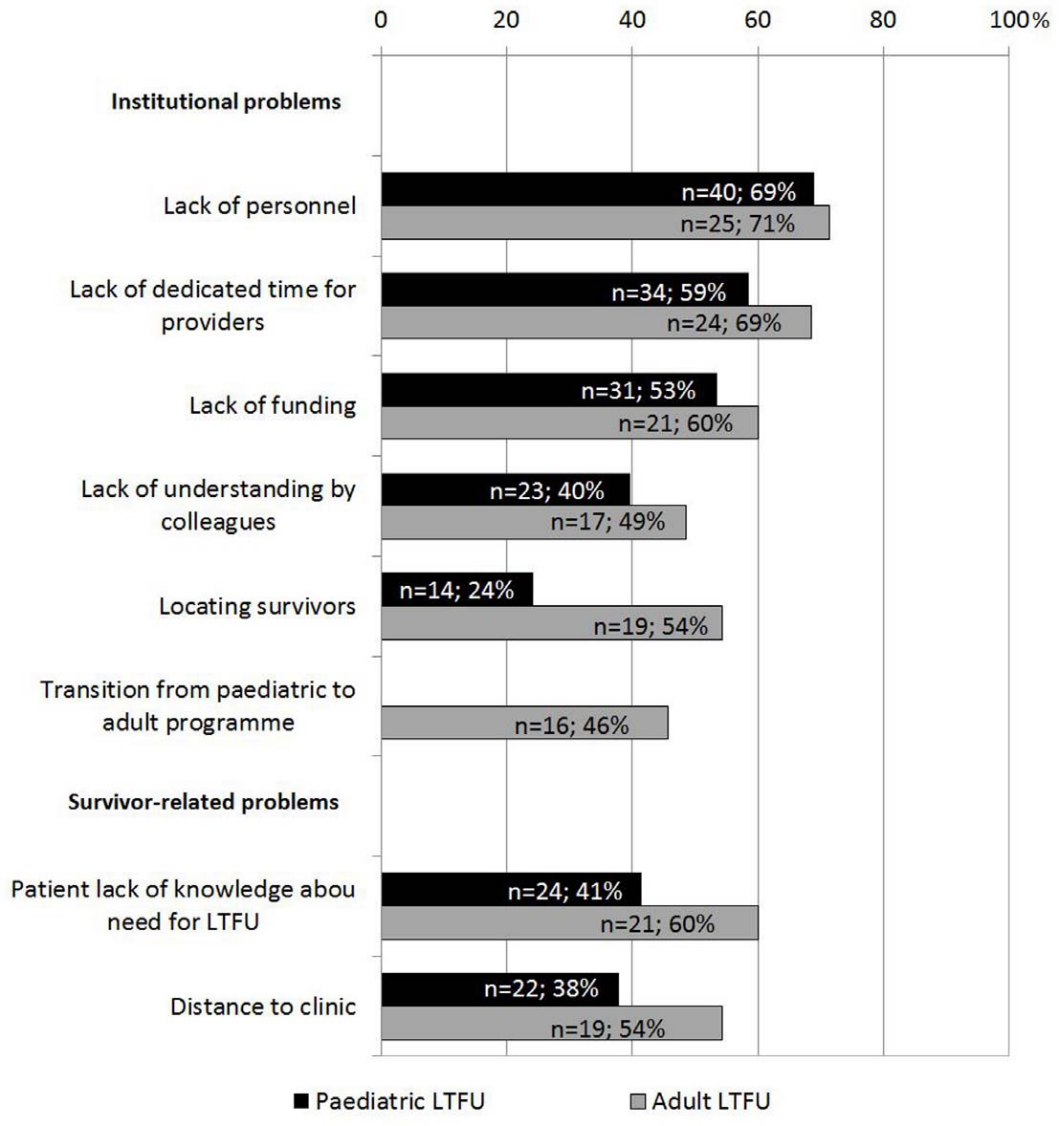
Problems encountered in LTFU. LTFU: long-term follow-up program.

The most frequently reported problems in LTFU for pediatric survivors were on the provider side (**Figure 4**). Problems on the survivor side were less frequent but still concerned a considerable number of institutions. There was no difference between European regions (all p>0.05). Detailed results are presented in **Table S2**. In LTFU for adult survivors, all problems were more common than in LTFU for pediatric survivors, but especially survivor-related problems.

### Optimal Care

#### Staff

When asked about who should ideally care for survivors most respondents agreed that a pediatric oncologist should be involved in LTFU for pediatric (n = 57, 100%) and adult survivors (n = 25, 81%; **Figure 1**). However, in addition medical oncologists, specialist nurses, general practitioners and social workers were desired often but not available for LTFU.

#### Organizational model

**Figure 5:**
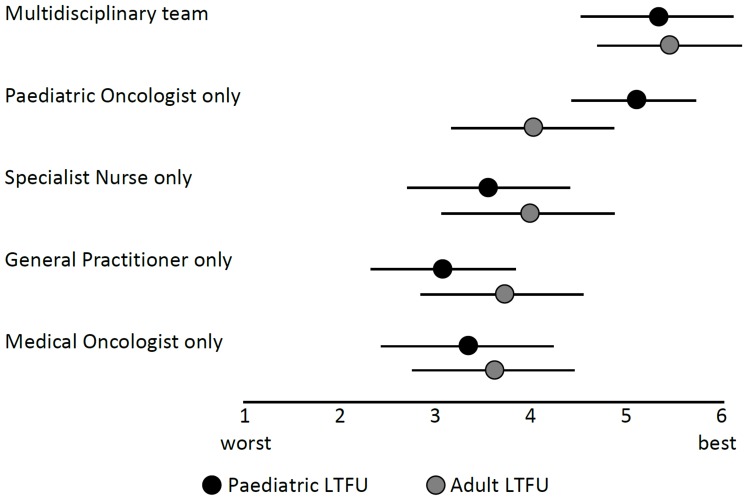
Rating of organizational models of care, by pediatric and adult LTFU. LTFU: long-term follow-up program; dots represent means and lines +/−1 standard deviation; Adapted from Wallace et al. [19].

Follow-up by a multidisciplinary team was rated higher than the other theoretical models of LTFU proposed for both pediatric and adult survivors (mean = 5.3, SD = 1.6 and mean = 5.4, SD = 1.5, respectively). Other proposed models were: pediatric oncologist (mean = 5.2, SD = 1.3; mean = 4.0, SD = 1.7), specialist nurse (mean = 3.6, SD = 1.7; mean = 4.0, SD = 1.8), medical oncologist (mean = 3.4, SD = 1.8; mean = 3.7, SD = 1.7), and general practitioner (mean = 3.1, SD = 1.5; mean = 3.8, SD = 1.7) (**Figure 5**). In LTFU for pediatric survivors, there were significant differences between European regions in their rating of the multidisciplinary team (F(4,54) = 3.62; p = 0.011) and nurse-led care (F(4,54) = 5.60; p<0.001). The multidisciplinary team was rated lowest in the Nordic countries, and nurse-led care was rated high only in the British Isles. Detailed results on the level of countries are presented in **Table S3**.

## Discussion

Our overview of follow-up programs showed that there is still a lack of LTFU for childhood cancer survivors in Europe. While 66% of institutions report LTFU for their pediatric survivors, only 38% have a LTFU available for adult survivors. However, even institutions with established LTFU also reported a number of institution- and survivor-related problems, particularly in programs for adult survivors. Most (86%) of the institutions without LTFU would like to provide this form of follow-up care to their survivors.

Surveys among childhood cancer survivors in Europe and elsewhere indicate that only some survivors receive high quality follow-up care [20]–[23]. LTFU does not necessarily equate to high quality care, and less formal LTFU might provide high quality care to individual survivors. However, well-organized LTFU will become more important with the ever increasing number of childhood cancer survivors.

The availability of LTFU across European regions varied between 9% and 83%. This could have different reasons. Access to funding, space and trained professionals with dedicated time for LTFU may differ across institutions and countries. On the British Isles we found a high proportion of institutions that provided LTFU. National guidelines available in England and Scotland [3], [6], [7], [24] may provide guidance and support to institutions wishing to provide LTFU in a formal setting.

### Comparison with other Studies

The availability of LTFU for pediatric survivors in Europe (66%) is comparable to the USA and Canada, but is lower for adult survivors (38%). A recent study reported that 59% of institutions of the COG, predominantly in the USA, had LTFU for pediatric and 47% for adult survivors [13]. In an earlier study in the USA on programs for adult survivors, 44% of institutions stated they had a program available [25]. In Canada, the respective proportions were >80% and 53% [14], [26].

We found that pediatric oncologists are running LTFU for pediatric and adult survivors in most institutions, supporting previous findings from USA and Canada [14], [26], [27]. Respondents agreed that pediatric oncologists should be involved in LTFU. This is in contrast with results from a study of pediatric oncologists in the USA, where only 38% reported that they wanted to be the doctor of a survivor for “as long as possible” [28].

Specialist nurses were available in all comprehensive follow-up programs described by Aziz and colleagues [27], and in most of the institutions (72%) participating in a recent survey among the COG [13]. For Europe, we found that less than half institutions routinely involved nurses in their program. Indeed, lack of a dedicated nurse practitioner was listed as a major disadvantage by respondents to our survey.

We asked respondents to rate different models of follow-up adapted from Wallace and colleagues [19]. Our data showed that GP- or nurse-led follow-up, which could be suitable for survivors with a low risk for late effects, were considerably less favored than follow-up by a multidisciplinary team, or by a pediatric oncologist in LTFU for pediatric survivors. However, a combination of models for survivors according to their risk for late effects might represent a future solution to many of the problems reported in our survey.

Most programs use follow-up guidelines, but around one in four reported providing little or no patient education about previous illness, treatment, late effects or health behavior. This might be a reason for attrition in follow up care. The proportion using guidelines was similar to that reported in a Canadian study (88%) [26]. Despite recommendations [29]–[31], provision of treatment summaries was relatively low: fewer than two thirds of all institutions with programs provide such a summary. This is similar to findings from France [32] and is especially notable because studies have found that survivors are relatively ignorant about their former disease and treatment [33]–[35]. Survivors are often unaware that follow-up is necessary. This lack of knowledge has been reported to be a major contributor to limited follow-up attendance [21]. Medical screening was a frequent activity during consultations, but provision of education of and information to survivors was less frequent and similar to findings from Canada [26]. This might not be sufficient, especially when considering survivors’ need for information and education [36], [37].

### Strengths and Limitations

This is the first multi-country overview of follow-up programs in Europe, and by far the most comprehensive. We provided detailed results on region and country level, allowing the reader to understand the considerable variability across Europe. We were able to contact almost all institutions treating children with cancer in Europe. However, we were not able to include data from two large countries (Germany and France) and some smaller countries not participating in PanCare, but information on follow-up care was recently assessed in France [17], [32] and should soon be available for Germany.

Self-report questionnaires have some inherent weaknesses. Because the survey was based on a self-report questionnaire, social desirability may have played a role in the description of the respondent’s own LTFU. Similarly, the nature of LTFU was self-categorized, although we gave a specific definition in the questionnaire. Participants’ estimated percentages have to be interpreted with caution and should not be understood as exact values in clinical practice.

### Conclusion

Long-term follow-up for childhood cancer patients is a necessary part of care for most childhood cancer patients after completion of treatment. Well-organized follow-up programs improve the quality of care for the increasing number of survivors. Our study showed that many European countries do not provide enough LTFU. Despite general agreement on the need for LTFU, and the existence of relevant guidelines, three of five European institutions do not implement such programs for adult survivors, and one of three does not provide them for pediatric survivors of childhood cancer. This is a potential disservice to former patients. Thanks to close international collaboration of pediatric oncologists and other specialists, as practiced in the PanCare Network and the PanCare SurFup project (www.pancare.eu), European guidelines for LTFU will soon be available. As in the British Isles, institutions may then profit from available knowledge and experience, and build their own program according to their needs and those of their survivors.

## Supporting Information

Table S1 **Content of pediatric and adult LTFU, by region and country.**
(DOCX)

Table S2 **Problems encountered in pediatric and adult LTFU, by region and country.**
(DOCX)

Table S3 **Rating of organizational models of care, by region and country.**
(DOCX)

Questionnaire S1 **Follow-up care after childhood cancer in Europe.**
(PDF)
