# Supplementary material for: Follow-Up Programs for Childhood Cancer Survivors in Europe: A Questionnaire Survey
Source: PLoS One. 2012 Dec 31;7(12):e53201. doi: 10.1371/journal.pone.0053201 (PMC3534070; doi:10.1371/journal.pone.0053201)
Supplement: Table S1 — Content of pediatric and adult LTFU, by region and country. (DOCX) [file pone.0053201.s001.docx]

**Online Supplementary to Manuscript “Follow-up for childhood cancer survivors in Europe: a questionnaire study”**

**Online Supplemental Table S1:** Content of pediatric and adult LTFU, by region and country

**Online Supplemental Table S1:** Content of pediatric and adult LTFU, by region and country

| **Region** | **Country** | **Screening for** | | | | | | | | **Education of survivors regarding** | | | | | | | |
| --- | --- | --- | --- | --- | --- | --- | --- | --- | --- | --- | --- | --- | --- | --- | --- | --- | --- |
|  |  | **Cancer recurrence** | | **Late effects** | | **Second malignant neoplasms** | | **Psychosocial problems** | | **Previous disease** | | **Treatment** | | **Potential future health problems** | | **Health behaviors** | |
|  |  | **Pediatric**  **n (%)** | **Adult**  **n (%)** | **Pediatric**  **n (%)** | **Adult**  **n (%)** | **Pediatric**  **n (%)** | **Adult**  **n (%)** | **Pediatric**  **n (%)** | **Adult**  **n (%)** | **Pediatric**  **n (%)** | **Adult**  **n (%)** | **Pediatric**  **n (%)** | **Adult**  **n (%)** | **Pediatric**  **n (%)** | **Adult**  **n (%)** | **Pediatric**  **n (%)** | **Adult**  **n (%)** |
| **British Isles** |  | **8 (80)** | **6 (86)** | **10 (100)** | **7 (100)** | **10 (100)** | **7 (100)** | **10 (100)** | **7 (100)** | **10 (100)** | **7 (100)** | **10 (100)** | **7 (100)** | **10 (100)** | **7 (100)** | **10 (100)** | **7 (100)** |
|  | Ireland | 1 (100) |  | 1 (100) |  | 1 (100) |  | 1 (100) |  | 1 (100) |  | 1 (100) |  | 1 (100) |  | 1 (100) |  |
|  | UK | 7 (78) | 6 (86) | 9 (100) | 7 (100) | 9 (100) | 7 (100) | 9 (100) | 7 (100) | 9 (100) | 7 (100) | 9 (100) | 7 (100) | 9 (100) | 7 (100) | 9 (100) | 7 (100) |
| **Northern Europe** |  | **6 (86)** | **1 (100)** | **7 (100)** | **1 (100)** | **6 (86)** | **1 (100)** | **7 (100)** | **1 (100)** | **7 (100)** | **1 (100)** | **7 (100)** | **1 (100)** | **7 (100)** | **1 (100)** | **6 (86)** | **1 (100)** |
|  | Denmark | 0 (0) |  | 1 (100) |  | 1 (100) |  | 1 (100) |  | 1 (100) |  | 1 (100) |  | 1 (100) |  | 1 (100) |  |
|  | Finland | 3 (100) |  | 3 (100) |  | 3 (100) |  | 3 (100) |  | 3 (100) |  | 3 (100) |  | 3 (100) |  | 3 (100) |  |
|  | Lithuania |  |  |  |  |  |  |  |  |  |  |  |  |  |  |  |  |
|  | Norway |  |  |  |  |  |  |  |  |  |  |  |  |  |  |  |  |
|  | Sweden | 3 (100) | 1 (100) | 3 (100) | 1 (100) | 2 (67) | 1 (100) | 3 (100) | 1 (100) | 3 (100) | 1 (100) | 3 (100) | 1 (100) | 3 (100) | 1 (100) | 2 (67) | 1 (100) |
| **Southern Europe** |  | **18 (90)** | **12 (86)** | **18 (90)** | **12 (86)** | **18 (90)** | **12 (86)** | **17 (85)** | **13 (93)** | **13 (65)** | **9 (64)** | **13 (65)** | **9 (64)** | **15 (75)** | **10 (71)** | **14 (70)** | **9 (64)** |
|  | Greece | 2 (67) | 1 (50) | 3 (100) | 2 (100) | 3 (100) | 1 (50) | 3 (100) | 2 (100) | 2 (67) | 1 (50) | 2 (67) | 1 (50) | 2 (67) | 1 (50) | 2 (67) | 1 (50) |
|  | Italy | 9 (90) | 8 (89) | 8 (80) | 7 (78) | 8 (80) | 8 (89) | 8 (80) | 8 (89) | 7 (70) | 6 (67) | 8 (80) | 7 (78) | 7 (70) | 7 (78) | 6 (60) | 6 (67) |
|  | Slovenia | 1 (100) | 1 (100) | 1 (100) | 1 (100) | 1 (100) | 1 (100) | 1 (100) | 1 (100) | 1 (100) | 1 (100) | 1 (100) | 1 (100) | 1 (100) | 1 (100) | 1 (100) | 1 (100) |
|  | Spain | 6 (100) | 2 (100) | 6 (100) | 2 (100) | 6 (100) | 2 (100) | 5 (83) | 2 (100) | 3 (50) | 1 (50) | 2 (33) | 0 (0) | 5 (83) | 1 (100) | 5 (83) | 1 (50) |
| **Western Europe** |  | **11 (92)** | **6 (86)** | **12 (100)** | **7 (100)** | **12 (100)** | **7 (100)** | **11 (92)** | **7 (100)** | **9 (75)** | **5 (71)** | **8 (67)** | **5 (71)** | **10 (83)** | **6 (86)** | **9 (75)** | **7 (100)** |
|  | Austria | 1 (100) | 1 (100) | 1 (100) | 1 (100) | 1 (100) | 1 (100) | 1 (100) | 1 (100) | 1 (100) | 1 (100) | 1 (100) | 1 (100) | 1 (100) | 1 (100) | 1 (100) | 1 (100) |
|  | Belgium | 2 (100) | 1 (100) | 2 (100) | 2 (100) | 2 (100) | 1 (100) | 2 (100) | 1 (100) | 2 (100) | 1 (100) | 2 (100) | 1 (100) | 2 (100) | 1 (100) | 1 (50) | 1 (100) |
|  | Netherlands | 3 (75) | 3 (75) | 4 (100) | 4 (100) | 4 (100) | 4 (100) | 4 (100) | 4 (100) | 3 (75) | 3 (100) | 3 (75) | 3 (75) | 4 (100) | 4 (100) | 4 (100) | 4 (100) |
|  | Switzerland | 5 (100) | 1 (100) | 5 (100) | 5 (100) | 5 (100) | 1 (100) | 4 (80) | 1 (100) | 3 (60) | 0 (0) | 2 (40) | 0 (0) | 3 (60) | 0 (0) | 3 (60) | 1 (100) |
| **Eastern Europe** |  | **8 (100)** | **2 (100)** | **8 (100)** | **2 (100)** | **8 (100)** | **2 (100)** | **7 (88)** | **2 (100)** | **5 (63)** | **2 (100)** | **5 (63)** | **2 (100)** | **7 (88)** | **2 (100)** | **5 (63)** | **2 (100)** |
|  | Czech Republic | 1 (100) | 1 (100) | 1 (100) | 1 (100) | 1 (100) | 1 (100) | 1 (100) | 1 (100) | 1 (100) | 1 (100) | 1 (100) | 1 (100) | 1 (100) | 1 (100) | 1 (100) | 1 (100) |
|  | Hungary | 2 (100) |  | 2 (100) |  | 2 (100) |  | 2 (100) |  | 1 (50) |  | 1 (50) |  | 2 (100) |  | 1 (50) |  |
|  | Poland | 4 (100) | 1 (100) | 4 (100) | 1 (100) | 4 (100) | 1 (100) | 3 (75) | 1 (100) | 2 (50) | 1 (100) | 2 (50) | 1 (100) | 3 (75) | 1 (100) | 2 (50) | 1 (100) |
|  | Slovak Republic | 1 (100) |  | 1 (100) |  | 1 (100) |  | 1 (100) |  | 1 (100) |  | 1 (100) |  | 1 (100) |  | 1 (100) |  |
| **Total** |  | **51 (89)** | **25 (78)** | **55 (96)** | **29 (91)** | **54 (95)** | **28 (87)** | **52 (91)** | **27 (84)** | **44 (77)** | **21 (66)** | **43 (75)** | **21 (66)** | **49 (86)** | **24 (75)** | **44 (77)** | **25 (78)** |

Abbreviations: LTFU, long-term follow-up program; Pediatric, Long-term follow-up program for pediatric survivors; Adult, Long-term follow-up program adult survivors of childhood cancer; na, not applicable
Empty fields indicate no answers to the respective question
