# Supplementary material for: Follow-Up Programs for Childhood Cancer Survivors in Europe: A Questionnaire Survey
Source: PLoS One. 2012 Dec 31;7(12):e53201. doi: 10.1371/journal.pone.0053201 (PMC3534070; doi:10.1371/journal.pone.0053201)
Supplement: Table S2 — Problems encountered in pediatric and adult LTFU, by region and country. (DOCX) [file pone.0053201.s002.docx]

**Online Supplementary to Manuscript “Follow-up for childhood cancer survivors in Europe: a questionnaire study”**

**Online Supplemental Table S2:** Problems encountered in pediatric and adult LTFU, by region and country

**Online Supplemental Table S2:** Problems encountered in pediatric and adult LTFU, by region and country

| **Region** | **Country** | **Institutional problems** | | | | | | | | | | | | **Survivor-related problems** | | | |
| --- | --- | --- | --- | --- | --- | --- | --- | --- | --- | --- | --- | --- | --- | --- | --- | --- | --- |
|  |  | **Lack of staff** | | **Lack of dedicated time for providers** | | **Lack of funding** | | **Lack of understanding by colleagues** | | **Problems locating survivors** | | **Transition from pediatric to adult program** | | **Patient lack of knowledge about need for LTFU** | | **Distance to clinic** | |
|  |  | **Pediatric**  **n (%)** | **Adult**  **n (%)** | **Pediatric**  **n (%)** | **Adult**  **n (%)** | **Pediatric**  **n (%)** | **Adult**  **n (%)** | **Pediatric**  **n (%)** | **Adult**  **n (%)** | **Pediatric**  **n (%)** | **Adult**  **n (%)** | **Pediatric**  **n (%)** | **Adult**  **n (%)** | **Pediatric**  **n (%)** | **Adult**  **n (%)** | **Pediatric**  **n (%)** | **Adult**  **n (%)** |
| **British Isles** |  | **6 (60)** | **6 (75)** | **6 (60)** | **7 (88)** | **5 (50)** | **6 (75)** | **6 (60)** | **5 (63)** | **5 (50)** | **6 (75)** | **na** | **2 (25)** | **5 (50)** | **6 (75)** | **5 (50)** | **7 (88)** |
|  | Ireland | 1 (100) |  | 1 (100) |  | 1 (100) |  | 0 (0) |  | 0 (0) |  | na |  | 0 (0) |  | 0 (0) |  |
|  | UK | 5 (56) | 6 (75) | 5 (56) | 7 (88) | 4 (44) | 6 (75) | 6 (67) | 5 (63) | 5 (56) | 6 (75) | na | 2 (25) | 5 (56) | 6 (75) | 5 (56) | 7 (88) |
| **Northern Europe** |  | **5 (71)** | **1 (100)** | **4 (57)** | **1 (100)** | **2 (29)** | **1 (100)** | **1 (14)** | **1 (100)** | **0 (0)** | **1 (100)** | **na** | **1 (100)** | **0 (0)** | **1 (100)** | **4 (57)** | **1 (100)** |
|  | Denmark | 0 (0) |  | 0 (0) |  | 1 (100) |  | 0 (0) |  | 0 (0) |  | na |  | 0 (0) |  | 0 (0) |  |
|  | Finland | 2 (67) |  | 1 (33) |  | 1 (33) |  | 0 (0) |  | 0 (0) |  | na |  | 0 (0) |  | 1 (33) |  |
|  | Lithuania |  |  |  |  |  |  |  |  |  |  | na |  |  |  |  |  |
|  | Norway |  |  |  |  |  |  |  |  |  |  | na |  |  |  |  |  |
|  | Sweden | 3 (100) | 1 (100) | 3 (100) | 1 (100) | 0 (0) | 1 (100) | 1 (33) | 1 (100) | 0 (0) | 1 (100) | na | 1 (100) | 0 (0) | 1 (100) | 3 (100) | 1 (100) |
| **Southern Europe** |  | **13 (65)** | **9 (64)** | **8 (40)** | **8 (57)** | **9 (45)** | **6 (43)** | **8 (40)** | **5 (36)** | **5 (25)** | **8 (57)** | **na** | **8 (57)** | **8 (40)** | **8 (57)** | **8 (40)** | **7 (50)** |
|  | Greece | 3 (100) | 1 (50) | 1 (33) | 1 (50) | 2 (67) | 1 (50) | 1 (33) | 1 (50) | 2 (67) | 2 (100) | na | 7 (78) | 0 (0) | 2 (100) | 2 (67) | 2 (100) |
|  | Italy | 6 (60) | 6 (67) | 5 (50) | 6 (67) | 6 (60) | 5 (56) | 5 (50) | 3 (33) | 3 (30) | 6 (67) | na | 0 (0) | 5 (50) | 5 (56) | 4 (40) | 5 (56) |
|  | Slovenia | 1 (100) | 1 (100) | 0 (0) | 0 (0) | 0 (0) | 0 (0) | 1 (100) | 1 (100) | 0 (0) | 0 (0) | na | 0 (0) | 1 (100) | 1 (100) | 0 (0) | 0 (0) |
|  | Spain | 3 (50) | 1 (50) | 2 (33) | 1 (50) | 1 (17) | 0 (0) | 1 (17) | 0 (0) | 0 (0) | 0 (0) | na | 1 (50) | 2 (33) | 0 (0) | 2 (33) | 0 (0) |
| **Western Europe** |  | **10 (83)** | **6 (100)** | **9 (75)** | **5 (83)** | **9 (75)** | **5 (83)** | **5 (42)** | **3 (50)** | **2 (17)** | **2 (33)** | **na** | **3 (50)** | **7 (58)** | **5 (83)** | **3 (25)** | **3 (50)** |
|  | Austria | 0 (0) | 1 (100) | 1 (100) | 1 (100) | 0 (0) | 0 (0) | 0 (0) | 0 (0) | 0 (0) | 0 (0) | na | 0 (0) | 1 (100) | 1 (100) | 0 (0) | 0 (0) |
|  | Belgium | 2 (100) | 1 (100) | 2 (100) | 1 (100) | 1 (50) | 1 (100) | 0 (0) | 1 (100) | 0 (0) | 0 (0) | na | 1 (100) | 1 (50) | 0 (0) | 1 (50) | 1 (100) |
|  | Netherlands | 4 (100) | 4 (100) | 2 (50) | 3 (75) | 4 (100) | 4 (100) | 2 (50) | 2 (50) | 1 (25) | 2 (50) | na | 2 (50) | 3 (75) | 4 (100) | 2 (50) | 2 (50) |
|  | Switzerland | 4 (80) |  | 4 (80) |  | 4 (80) |  | 3 (60) |  | 1 (20) |  | na |  | 2 (40) |  | 0 (0) |  |
| **Eastern Europe** |  | **5 (63)** | **1 (50)** | **6 (75)** | **1 (50)** | **5 (63)** | **1 (50)** | **3 (38)** | **1 (50)** | **2 (25)** | **1 (50)** | **na** | **0 (0)** | **3 (38)** | **0 (0)** | **2 (25)** | **1 (50)** |
|  | Czech Republic | 1 (100) | 0 (0) | 1 (100) | 0 (0) | 0 (0) | 0 (0) | 1 (100) | 0 (0) | 0 (0) | 0 (0) | na | 0 (0) | 0 (0) | 0 (0) | 0 (0) | 0 (0) |
|  | Hungary | 1 (50) |  | 1 (50) |  | 1 (50) |  | 1 (50) |  | 1 (50) |  | na |  | 1 (50) |  | 1 (50) |  |
|  | Poland | 3 (75) | 1 (100) | 3 (75) | 1 (100) | 4 (100) | 1 (100) | 1 (25) | 1 (100) | 1 (25) | 1 (100) | na | 0 (0) | 1 (25) | 0 (0) | 1 (25) | 1 (100) |
|  | Slovak Republic | 0 (0) |  | 1 (100) |  | 0 (0) |  | 0 (0) |  | 0 (0) |  | na |  | 1 (100) |  | 0 (0) |  |
| **Total** |  | **40 (69)** | **25 (71)** | **34 (59)** | **24 (69)** | **31 (53)** | **21 (60)** | **23 (40)** | **17 (49)** | **14 (24)** | **19 (54)** | **na** | **16 (46)** | **24 (41)** | **21 (60)** | **22 (38)** | **19 (54)** |

Abbreviations: LTFU, long-term follow-up program; Pediatric, Long-term follow-up program for pediatric survivors; Adult, Long-term follow-up program adult survivors of childhood cancer; na, not applicable
Empty fields indicate no answers to the respective question
