# Supplementary material for: Follow-Up Programs for Childhood Cancer Survivors in Europe: A Questionnaire Survey
Source: PLoS One. 2012 Dec 31;7(12):e53201. doi: 10.1371/journal.pone.0053201 (PMC3534070; doi:10.1371/journal.pone.0053201)
Supplement: Table S3 — Rating of organizational models of care, by region and country. (DOCX) [file pone.0053201.s003.docx]

**Online Supplementary to Manuscript “Follow-up for childhood cancer survivors in Europe: a questionnaire study”**

**Online Supplemental Table S3:** Rating of organizational models of care, by region and country

**Online Supplemental Table S3:** Rating of organizational models of care, by region and country

| **Region** | **Country** | **Multidisciplinary team** | | **Pediatric Oncologist only** | | **Specialist nurse only** | | **General practitioner only** | | **Medical oncologist only** | |
| --- | --- | --- | --- | --- | --- | --- | --- | --- | --- | --- | --- |
|  |  | **Pediatric**  **mean (SD)** | **Adult**  **mean (SD)** | **Pediatric**  **mean (SD)** | **Adult**  **mean (SD)** | **Pediatric**  **mean (SD)** | **Adult**  **mean (SD)** | **Pediatric**  **mean (SD)** | **Adult**  **mean (SD)** | **Pediatric**  **mean (SD)** | **Adult**  **mean (SD)** |
| **British Isles** |  | **5.7 (0.9)** | **5.9 (0.4)** | **5.8 (0.4)** | **3.6 (1.6)** | **5.4 (0.8)** | **4.6 (1.7)** | **3.1 (1.7)** | **3.3 (1.6)** | **4 (1.9)** | **4.5 (1.7)** |
|  | Ireland | 6 (-) |  | 6 (-) |  | 6 (-) |  | 3 (-) |  | 5 (-) |  |
|  | UK | 5.7 (1) | 5.9 (0.4) | 5.8 (0.4) | 3.6 (1.6) | 5.3 (0.9) | 4.6 (1.7) | 3.1 (1.8) | 3.4 (1.6) | 3.9 () | 4.5 (1.7) |
| **Northern Europe** |  | **3.6 (2.5)** | **6 (-)** | **5 (1.8)** | **4 (-)** | **4 (1.9)** | **6 (-)** | **3 (1.5)** | **2 (-)** | **3.7 (1.8)** | **5 (-)** |
|  | Denmark | 4 (-) |  | 6 (-) |  | 5 (-) |  | 3 (-) |  | 2 (-) |  |
|  | Finland | 4.3 (2.9) |  | 4 (2.6) |  | 3 (1.7) |  | 3.3 (1.5) |  | 5.3 (0.6) |  |
|  | Lithuania |  |  |  |  |  |  |  |  |  |  |
|  | Norway |  |  |  |  |  |  |  |  |  |  |
|  | Sweden | 2.7 (2.9) | 6 (-) | 5.7 (0.6) | 4 (-) | 4.7 (2.3) | 6 (-) | 2.7 (2.1) | 2 (-) | 2.7 (1.5) | 5 (-) |
| **Southern Europe** |  | **5.7 (1.1)** | **5.5 (1.3)** | **5.4 (1.1)** | **4.2 (1.8)** | **2.8 (1.3)** | **3.5 (1.7)** | **3.4 (1.4)** | **4.4 (1.6)** | **3.2 (1.9)** | **3.4 (1.6)** |
|  | Greece | 4.3 (2.9) | 3 (2.8) | 5 (1.7) | 4.5 (2.1) | 4 (1) | 4 (1.4) | 2.3 (0.6) | 4 (1.4) | 5.3 (0.6) | 4 (1.4) |
|  | Italy | 5.9 (0.3) | 5.9 (0.3) | 5.6 (0.7) | 4.1 (1.6) | 3.1 (1.1) | 4 (1.5) | 3.9 (1.1) | 5.2 (0.8) | 2.7 (1.9) | 3.2 (1.7) |
|  | Slovenia | 6 (-) | 6 (-) | 2 (-) | 1 (-) | 1 (-) | 1 (-) | 1 (-) | 1 (-) | 2 (-) | 1 (-) |
|  | Spain | 6 (0) | 6 (-) | 5.7 (0.5) | 6 (0) | 2 (1.3) | 2 (1.4) | 3.3 (1.8) | 3 (1.4) | 3.1 (1.9) | 4.5 (0.7) |
| **Western Europe** |  | **5 (1.9)** | **4.2 (2.5)** | **4.3 (1.7)** | **3.3 (2.1)** | **3.7 (1.7)** | **3.7 (2.2)** | **3.4 (1.4)** | **3.3 (1.6)** | **2.75 (1.7)** | **2.7 (1.4)** |
|  | Austria | 6 (-) | 6 (-) | 6 (-) | 6 (-) | 6 (-) | 6 (-) | 3 (-) | 3 (-) | 2 (-) | 3 (-) |
|  | Belgium | 6 (0) | 6 (-) | 5 (0) | 4 (-) | 2.5 (2.1) | 1 (-) | 3.5 (2.1) | 2 (-) | 1.5 (0.7) | 4 (-) |
|  | Netherlands | 3.5 (2.9) | 3.3 (2.6) | 3 (1.8) | 2.5 (1.9) | 3.8 (1.9) | 3.8 (1.9) | 3 (1.4) | 3.8 (1.9) | 1.5 (1) | 2.3 (1.5) |
|  | Switzerland | 5.6 (0.5) |  | 4.6 (1.5) |  | 3.6 (1.5) |  | 3.8 (1.6) |  | 4.4 (1.1) |  |
| **Eastern Europe** |  | **6 (0)** | **6 (0)** | **5.4 (0.7)** | **5.5 (0.7)** | **3 (1.7)** | **5 (1.4)** | **2.3 (1.2)** | **4 (2.8)** | **3.5 (1.6)** | **5 (1.4)** |
|  | Czech Republic | 6 (-) | 6 (-) | 6 (-) | 6 (-) | 4 (-) | 6 (-) | 4 (-) | 6 (-) | 5 (-) | 6 (-) |
|  | Hungary | 6 (0) |  | 5 (1.4) |  | 3 (1.4) |  | 1 (-) |  | 2.5 (2.1) |  |
|  | Poland | 6 (0) | 6 (-) | 5.3 (0.5) | 5 (-) | 2.3 (1.9) | 4 (-) | 2.5 (1) | 2 (-) | 3.8 (1.7) | 4 (-) |
|  | Slovak Republic | 6 (-) |  | 6 (-) |  | 5 (-) |  | 2 (-) |  | 3 (-) |  |
| **Total** |  | **5.3 (1.6)** | **5.4 (1.5)** | **5.2 (1.3)** | **4.0 (1.7)** | **3.6 (1.7)** | **4.0 (1.8)** | **3.1 (1.5)** | **3.8 (1.7)** | **3.4 (1.8)** | **3.7 (1.7)** |

Abbreviations: LTFU, long-term follow-up program; Pediatric, Long-term follow-up program for pediatric survivors; Adult, Long-term follow-up program adult survivors of childhood cancer; SD, standard deviation; na, not applicable
Empty fields indicate no answers to the respective question
