# Supplementary material for: Follow-Up Programs for Childhood Cancer Survivors in Europe: A Questionnaire Survey
Source: PLoS One. 2012 Dec 31;7(12):e53201. doi: 10.1371/journal.pone.0053201 (PMC3534070; doi:10.1371/journal.pone.0053201)
Supplement: Questionnaire S1 — Follow-up care after childhood cancer in Europe. (PDF) [file pone.0053201.s004.pdf]

Instruction for questionnaire: Follow-Up Care after Childhood Cancer in Europe

What do we mean by follow-up care?

In this questionnaire we would like to assess how follow-up care for childhood cancer survivors is organised across Europe. We include separate sections (subsections 2A and 2B) to describe follow-up care for child and adult survivors of childhood cancer because follow-up care may differ widely.

A short definition before starting the questionnaire: With follow-up care we mean any type of care / appointments / examinations provided for childhood cancer survivors after they successfully finished treatment for cancer. Follow-up care might be irregular (or single) consultations with a paediatric oncologist, or it might include a formal well-organised follow-up care programme with regular scheduled visits for survivors.

The questionnaire contains three sections:

- **Section 1**: Information about the respondent and his/her institution
- **Section 2**: Follow-Up Care after Childhood Cancer in Europe
  (A*) "*Follow-Up Care Programme for Children*" and
  (B*) "*Follow-Up Care Programme for Adult Survivors of Childhood Cancer*"
- **Section 3***: Guidelines for Long-Term Follow-Up.

* You will be automatically guided through the questions relevant for your institution.

Thank you very much for your cooperation.

SECTION 1: Information about the Respondent

1. Your name and firstname:

Your family name ______________________________

Your first name ______________________________

2. Your centre or hospital:

Name of your centre or hospital ______________________________

3. Your City:

In which city is your center or hospital located? ______________________________

4. Your country:

In which country is your center or hospital located? ______________________________

5. Your job title:

(e.g. „Consultant in Paediatric Oncology“, „Specialist nurse“, etc.) ______________________________

6. Today's date:

mm/dd/yyyy ______________________________

7. Type of institution where you are working:

Please only tick your main department/ward.

	Paediatric Oncology / Haematology	Paediatric department	Adult ward
University hospital or similar	☐	☐	☐
Smaller hospital or similar	☐	☐	☐
Private hospital	☐	☐	☐
Other: (please specify) ____	☐	☐	☐

8. How many clinical staff are employed in the department/ward? Please give full time employment equivalents.

Please write „0" (zero) if there is no staff in a certain group.

Number of junior doctors	____
Number of consultants / senior doctors	____
Number of nurses	____
Number of psychologists	____
Number of social workers	____
Number of other staff	____

Please specify other staff ____

9. In which areas of care are you involved?

Please tick all that apply.

- ☐ Inpatient acute care of newly diagnosed patients (Inpatient clinic)
- ☐ Outpatient acute care of patients under treatment (Outpatient clinic)
- ☐ Short-term follow-up of patients who completed treatment (<5 years after diagnosis)
- ☐ Long-term follow-up of patients who completed treatment (> 5 years after diagnosis)
- ☐ Other: (please specify) ____

10. How many patients (not consults or second opinions) with newly-diagnosed childhood cancer were treated at your institution in the last 12 months?

About ____ new patients

11. What proportion of newly diagnosed patients (not relapse / recurrent disease) is included in clinical studies for treatment at your institution?

About ____ % of new patients

12. Are patients (or their parents) given a written summary of their cancer treatment at the end of their active treatment?

Just tick one check box!

- ☐ Yes, patients always receive a treatment summary
- ☐ Yes, patients with certain diagnoses receive a treatment summary (please list diagnoses) ____
- ☐ Yes, on patient's request
- ☐ Yes, but rarely
- ☐ Never

13. Are patients (or their parents) given information about late effects, or life after cancer?

Just tick one check box per column!

* **Patient specific** may contain any information about future screening or regularity of follow-up for each patient according to their cancer and treatment.

** **General leaflet** may contain any general information about the usefulness of screening, follow-up care or health behaviour for survivors in general.

	Patient specific *
Yes, patients always get information	☐
Yes, patients with certain diagnoses get information (please list diagnoses)	☐
Yes, on patient's request	☐
Yes, but rarely	☐
Never	☐

	General leaflet **
Yes, patients always get information	☐
Yes, patients with certain diagnoses get information (please list diagnoses)	☐
Yes, on patient's request	☐
Yes, but rarely	☐
Never	☐

Could you please send us an example of an anonymised patient-specific treatment summary and the information you give patients about follow-up care, late effects or life after cancer?

E-mail to:
michel@ispm.unibe.ch

...or mail to:
Gisela Michel, PhD
Institute of Social and Preventive Medicine
University of Bern
Finkenhubelweg 11
3012 Bern
Switzerland

SECTION 2: Follow-Up Care after Childhood Cancer in Europe

Important: Please answer these questions only in reference to the institution where you work.

14. Please describe briefly, how follow-up care is organised in your institution.

Please also mention differences in follow up care for survivors who had cancer at a very young age or in adolescence.

How are patients followed up after end of treatment?	
How are patients followed up when they reach adulthood (usually between 16 to 20 years of age)?	
What is follow-up care called at your institution (e.g. Late effects clinic, Multidisciplinary follow-up clinic, LTFU, ...)?	

Availability of follow-up

15. Do you have a formal follow-up programme* / follow-up clinic* dedicated for survivors of childhood cancer in your institution? (e.g. late effects clinic)

* As a formal follow-up programme or formal follow-up clinic we define a model of specialised care dedicated to follow-up childhood cancer survivors. Specialised care may contain comprehensive, evidence-based health care and education for survivors of childhood cancer. Follow-up only done for participants of clinical trials during trial follow-up should not be included in this definition.

Please answer all subquestions.

	Yes	No
Follow-up programme for children (survivors <16 years of age)	○	○
Follow-up programme for adult survivors (survivors >16 years of age)	○	○
Transition programme (specific programme to transfer survivors from paediatric to adult care)	○	○
Other (please describe „Other“ in the field below...)	○	○
There is no special programme, but paediatric oncologists/haematologists follow-up their former patients	○	○
No, we do not follow-up survivors at our institution	○	○

Please specify other	

Important: If you do NOT follow up survivors in your institution please continue on page 27.

16. Does follow-up provided at your institution only cover your institution, or are there patients referred from other institutions?

Please indicate the proportion of patients from your institution, and the proportion of patients referred from other institutions seperately. The proportions should add up to 100%.

Our institution		%
Patients referred from other institutions		%
Summe	---	%

17. If you have other institutions referring patients for follow-up (see previous question), please list name and place of each institution!

Example:

A) Clinic Lindenhof, Bern

B) Clinic Beau-Site, Bern

C) Hospital of Biel, Biel

A) Name of institution and place	
B) Name of institution and place	
C) Name of institution and place	
D) Name of institution and place	
E) Additional institutions	

Important: If you do NOT follow up **children** please skip this part and continue on page 18.

SECTION 2: Follow-Up Care after Childhood Cancer in Europe
A) Follow-Up Care Programme (or Clinic) for Children

Characteristics and routine activities of the follow-up clinic for children

In question 15 you answered that your institution has a formal follow-up programme / follow-up clinic for child and adolescent survivors of childhood cancer (i.e. survivors from early childhood until age 16 or 20 years).

Please refer to this programme when answering the questions in this section.

1. What age groups of survivors are included in your follow-up clinic?

From age ____ years to age ____ years

2. Survivors of which diagnoses are followed up?

Please tick all relevant for your institution.

- ☐ Leukaemias
- ☐ Lymphomas
- ☐ Brain tumours: – malign brain tumours
- ☐ Brain tumours: – benign brain tumours
- ☐ Neuroblastomas
- ☐ Retinoblastomas
- ☐ Nephroblastomas
- ☐ Hepatic tumours
- ☐ Bone tumours
- ☐ Soft tissue sarcomas
- ☐ Other tumours
- ☐ Transplant patients
- ☐ Other: (please specify) ____

3. What is the speciality of the head / director of the follow-up programme at your institution?

4. What is the speciality of the person who is routinely running the follow-up programme at your institution?

5. Is the paediatric oncologist / haematologist who treated the patient's cancer routinely involved in follow-up care?

- ○ Yes
- ○ No

6. Who is additionally routinely involved in follow-up care at your institution?

Please tick **all relevant** for your institution.

Do not tick specialities which are not routinely part of the follow-up programme and where you have to refer survivors separately.

- ☐ Paediatric oncologist/haematologist
- ☐ Designated general long-term follow-up physician
- ☐ Radiation oncologist
- ☐ Medical oncologist or haematologist (for adults)
- ☐ Specialist nurse
- ☐ Endocrinologist or paediatric endocrinologist
- ☐ Psychologist
- ☐ Neuropsychologist
- ☐ General practitioner or general paediatrician
- ☐ Social worker
- ☐ Other: (please specify)
- ☐ No other staff routinely involved.

7. In what setting is the follow-up programme situated?

Please tick **all relevant** for your institution.

- ☐ In the paediatric hospital or ward
- ☐ In an adult hospital or ward
- ☐ In another place: (please specify) ______

8. When do patients get transferred from the general paediatric oncology to the formal follow-up programme for young survivors of childhood cancer?

Please specify the number of years off treatment for each diagnosis separately.

Leukaemias		years off active treatment
Lymphomas		years off active treatment
Brain tumours: – malign brain tumours		years off active treatment
Brain tumours: – benign brain tumours		years off active treatment
Neuroblastomas		years off active treatment
Retinoblastomas		years off active treatment
Nephroblastomas		years off active treatment
Hepatic tumours		years off active treatment
Bone tumours		years off active treatment
Soft tissue sarcomas		years off active treatment
Other tumours		years off active treatment
Transplant patients		years off active treatment
Other:		years off active treatment

Please specify other ______

9. How frequently does the follow-up programme take place?

Please select one possibility.

- ☐ Every day
- ☐ Every week
- ☐ Every two weeks
- ☐ No regular follow-up clinic but can be arranged at patient's request
- ☐ Other: (please describe) ______

10. Are survivors reminded to attend follow-up clinic?

If you have different possibilities, please briefly decribe which possibility is used for a certain patient group.

- ☐ Yes, survivors receive an appointment
- ☐ Yes, survivors receive an invitation to contact the clinic for an appointment
- ☐ No, survivors are not reminded, and if they want to attend they contact the clinic themselves
- ☐ Other, please describe. ______

11. How many childhood cancer survivors (<16 years) attended follow-up in the past 12 months at your institutions

About ______ survivors

12. What proportion of survivors is attending followed-up?

Please estimate for each diagnosis and for time after diagnosis separately the proportion of survivors who are attending follow-up in your institution. If you cannot tell it exactly, please make a rough guess.

Example:

95% of leukaemia survivors <5 yrs after dx (i.e. 5% of leukaemia survivors <5 years after dx do not attend),
65% of lymphoma survivors who are between 5-10 years after dx (i.e. 35% of lymphoma survivors 5-10 years after dx do not attend),
0% of benign brain tumour survivors who are more than 10 years after diagnosis (i.e. 100% of survivors of benign brain tumour >10 years after dx do not attend)

Years after diagnosis	<5 years	5-10 years	>10 years
Leukaemias (Example)	95 %	70 %	50 %
Lymphomas(Example)	90 %	65 %	45 %
Brain tumours (benign) (Example)	50 %	30 %	0 %

Years after diagnosis

	<5 years	5-10 years	>10 years
Leukaemias			
Lymphomas			
Brain tumours: – malign brain tumours			
Brain tumours: – benign brain tumours			
Neuroblastomas			
Retinoblastomas			
Nephroblastomas			
Hepatic tumours			
Bone tumours			
Soft Tissue Sarcomas			
Other tumours			
Transplant patients			
Other			

Please specify other

13. Do you discharge survivors eventually?

Please tick all that apply.

- ☐ Yes, to a transition programme (a programme dedicated to ease transfer from paediatric to adult care): (please describe briefly)
- ☐ Yes, to a medical oncologist or haematologist (for adults)
- ☐ Yes, to general practitioner or general paediatrician
- ☐ Yes, other: (please describe briefly)
- ☐ No, we do not officially discharge survivors from our follow-up programme

14. What are the main reasons for discharging survivors?

Please list the most important reason first, and then other reasons, in order of importance.

1)
2)
3)
4)
5)
6)
7)
8)

15. What activities are routinely included in your follow-up programme?

Please tick all that apply.

- [ ] Check for cancer recurrence
- [ ] Check for late effects
- [ ] Check for second malignant neoplasms
- [ ] Check for psychosocial problems
- [ ] Educate survivors about their former disease (the cancer)
- [ ] Educate survivors about treatment they received
- [ ] Educate survivors about potential future health problems (late effects)
- [ ] Educate survivors about health behaviours (smoking, drinking, sun protection, etc)
- [ ] Other: (please specifiy)

SECTION 2: Follow-Up Care after Childhood Cancer in Europe
A) Follow-Up Care Programme (or Clinic) for Children

Advantages and disadvantages of the follow-up clinic at your institution

1. What are the advantages of the follow-up programme as it is organised at your institution?

1)
2)
3)
4)
5)
6)
7)
8)

2. What are the disadvantages of the follow-up programme as it is organised at your institution?

1)
2)
3)
4)
5)
6)
7)
8)

3. What kind of problems / barriers do you encounter with the follow-up clinic at your institution?

Please answer all subquestions.

	Yes	No
Patients' lack of knowledge about the need for follow-up	○	○
Inability to locate survivors	○	○
Distance to clinic (too far for survivors to come to clinic)	○	○
Lack of funding	○	○
Lack of dedicated time for providers	○	○
Lack of understanding by colleagues	○	○
Lack of personnel	○	○
Other problems: (If you tick „Yes" please specify below.)	○	○

Please specify other

4. In your opinion, who should care for young long-term survivors of childhood cancer?

Please answer all subquestions.

	Yes	No
Paediatric oncologist	○	○
Medical oncologist or haematologist (for adults)	○	○
Specialist nurse	○	○
Endocrinologist	○	○
Psychologist / Neuropsychologist	○	○
General practitioner	○	○
Social worker	○	○
Other: (If you tick „Yes" please specify below.)	○	○

Please specify other

5. In your opinion, what would be the optimal model of follow-up care for young survivors for your institution?

Please rank all models of follow up care according to your opinion from 1 to 6 (1 is the most optimal model and 6 the least optimal).

	1	2	3	4	5	6
Paediatric oncologist follows up childhood cancer survivors	○	○	○	○	○	○
Medical oncologist or haematologist follows up childhood cancer survivors	○	○	○	○	○	○
Multidisciplinary Team	○	○	○	○	○	○
Transition to general practitioner or general paediatrician	○	○	○	○	○	○
Specialist nurse follows up childhood cancer survivors	○	○	○	○	○	○

Any other model you would like to rank?

Important: If you do **not** follow up **adults survivors** please skip this part and continue on page 27.

SECTION 2: Follow-Up Care after Childhood Cancer in Europe
B) Follow-Up Programme for Adults

Characteristics and routine activities of the follow-up clinic for ADULTS

Please answer the following questions only if your institution has a formal follow-up programme dedicated to ADULT survivors of childhood cancer (i.e. survivors of childhood cancer older than 16 to 20 years).

1. From what age are childhood cancer survivors attending the follow-up programme for ADULT survivors of childhood cancer?

From age ______ years

2. Survivors of which diagnoses are followed up in the follow-up programme for adult survivors?

Please **tick all** relevant for your institution.

- ☐ Leukaemias
- ☐ Lymphomas
- ☐ Brain tumours: – malign brain tumours
- ☐ Brain tumours: – benign brain tumours
- ☐ Neuroblastomas
- ☐ Retinoblastomas
- ☐ Nephroblastomas
- ☐ Hepatic tumours
- ☐ Bone tumours
- ☐ Soft tissue sarcomas
- ☐ Other tumours
- ☐ Transplant patients
- ☐ Other: (please specify) ______

3. What is the speciality of the head / director of the follow-up programme at your institution?

4. What is the speciality of the person who is routinely running the follow-up programme at your institution?

5. Is the paediatric oncologist / haematologist who treated the patient's cancer routinely involved in follow-up care?

- ○ Yes
- ○ No

6. Who is additionally routinely involved in follow-up care for adult survivors at your institution

Please tick all relevant for your institution.

Do not tick specialities if you have to refer survivors specifically and which are not routinely part of the follow-up programme.

- ☐ Paediatric oncologist/haematologist
- ☐ Designated general long-term follow-up physician
- ☐ Radiation oncologist
- ☐ Medical oncologist or haematologist (for adults)
- ☐ Specialist nurse
- ☐ Endocrinologist or paediatric endocrinologist
- ☐ Psychologist
- ☐ Neuropsychologist
- ☐ General practitioner or general paediatrician
- ☐ Social worker
- ☐ Other: (please specify)
- ☐ No other staff routinely involved.

7. In what setting is the follow-up programme for adult survivors situated?

Please tick all relevant for your institution.

- ☐ In the paediatric hospital or ward
- ☐ In an adult hospital or ward
- ☐ In another place: (please specify) ____________

8. How frequently does the follow-up programme take place?

Please select one possibility.

- ☐ Every day
- ☐ Every week
- ☐ Every two weeks
- ☐ No regular follow-up clinic but can be arranged at patient's request
- ☐ Other: (please describe) ____________

9. Are survivors reminded to attend follow-up clinic?

If you have different possibilities, please briefly describe.

- ☐ Yes, survivors receive an appointment
- ☐ Yes, survivors receive an invitation to contact the clinic for an appointment
- ☐ No, survivors are not reminded, and if they want to attend they contact the clinic themselves
- ☐ Other, please describe. ____________

10. How many adult childhood cancer survivors attended follow-up in the past 12 months at your institution?

About ______ survivors

11. What proportion of adult survivors is attending followed-up?

Please estimate for each diagnosis and for time after diagnosis separately the proportion of survivors who are attending follow-up in your institution. If you cannot tell it exactly, please make a rough guess.

***Example**:*

95% of leukaemia survivors <5 yrs after dx (i.e. 5% of leukaemia survivors <5 years after dx do not attend),
65% of lymphoma survivors who are between 5-10 years after dx (i.e. 35% of lymphoma survivors 5-10 years after dx do not attend),
0% of benign brain tumour survivors who are more than 10 years after diagnosis (i.e. 100% of survivors of benign brain tumour >10 years after dx do not attend)

Age at follow-up	<5 years	5-10 years	>10 years
Leukaemias (Example)	95 %	70 %	50 %
Lymphomas(Example)	90 %	65 %	45 %
Brain tumours (benign) (Example)	50 %	30 %	0 %

Age at follow-up!

	<5 years	5-10 years	>10 years
Leukaemias			
Lymphomas			
Brain tumours: – malign brain tumours			
Brain tumours: – benign brain tumours			
Neuroblastomas			
Retinoblastomas			
Nephroblastomas			
Hepatic tumours			
Bone tumours			
Soft Tissue Sarcomas			
Other tumours			
Transplant patients			
Other			

Please specify others

12. Until what age do you follow-up adult survivors of childhood cancer in your follow-up clinic?

Until age [ ] years

13. Until how many years after end of treatment do you follow-up adult survivors of childhood cancer in your follow-up clinic?

Until [ ] years after end of treatment

14. Do you discharge survivors eventually?

Please tick all that apply.

- ☐ Yes, to a medical oncologist or haematologist (for adults)
- ☐ Yes to general practitioner
- ☐ Yes, other: (please describe briefly) [ ]
- ☐ No, we do not officially discharge survivors from our follow-up programme

15. What are the main reasons for discharging survivors?

Please list the most important reason first, and then other reasons, in order of importance.

1) [ ]
2) [ ]
3) [ ]
4) [ ]
5) [ ]
6) [ ]
7) [ ]
8) [ ]

16. What activities are routinely included in your follow-up programme?

Please tick all that apply.

- ☐ Check for cancer recurrence
- ☐ Check for late effects
- ☐ Check for second malignant neoplasms
- ☐ Check for psychosocial problems
- ☐ Educate survivors about their former disease (the cancer)
- ☐ Educate survivors about treatment they received
- ☐ Educate survivors about potential future health problems (late effects)
- ☐ Educate survivors about health behaviours (smoking, drinking, sun protection, etc)
- ☐ Other: (please specifiy)

SECTION 2: Follow-Up Care after Childhood Cancer in Europe
B) Follow-Up Programme for Adults

Advantages and disadvantages of the follow-up clinic for adult survivors at your institution

1. What are the advantages of the follow-up programme as it is organised at your institution?

1) ______
2) ______
3) ______
4) ______
5) ______
6) ______
7) ______
8) ______

2. What are the disadvantages of the follow-up programme as it is organised at your institution?

1) ______
2) ______
3) ______
4) ______
5) ______
6) ______
7) ______
8) ______

3. Please tick all problems / barriers that you encounter with the follow-up clinic for adult survivors at your institution?

Please answer all subquestion.

	Yes	No
Patients' lack of knowledge about the need for follow-up	○	○
Inability to locate survivors	○	○
Distance to clinic	○	○
Transition from paediatric care to adult follow-up programme	○	○
Survivors' lack of interest in continuing follow-up care in an adult programme	○	○
Lack of funding	○	○
Lack of dedicated time for providers	○	○
Lack of understanding by colleagues	○	○
Lack of personnel	○	○
Other problems: (If you tick „Yes" please specify below.)	○	○

Please specify other

4. In your opinion, who should care for adult long-term survivors of cancer?

Please answer all subquestion.

	Yes	No
Paediatric oncologist	○	○
Medical oncologist or haematologist (for adults)	○	○
Specialist nurse	○	○
Endocrinologist	○	○
Psychologist / Neuropsychologist	○	○
General practitioner	○	○
Social worker	○	○
Other: (If you tick „Yes" please specify below.)	○	○

Please specify other

5. In your opinion, what would be the optimal model of follow-up care for adult survivors of childhood cancer for your institution?

Please rank all models of follow up care according to your opinion from 1 to 6 (1 is the most optimal model and 6 the least optimal).

	1	2	3	4	5	6
Paediatric oncologist follows up adult survivors of childhood cancer	○	○	○	○	○	○
Medical oncologist or haematologist follows up adult survivors childhood cancer	○	○	○	○	○	○
Multidisciplinary team	○	○	○	○	○	○
Transition to general practitioner	○	○	○	○	○	○
Specialist nurse follows up childhood cancer survivors	○	○	○	○	○	○

Any other model you would like to rank?

Please continue on page 28.

No Follow-up programme for children and adults?

If you do not follow up survivors at your institution, do you send survivors somewhere else for follow-up?

- ○ Yes
- ○ No

Where do you send survivors for follow-up care?

Please write name or describe.

	Yes	No
Another hospital or external specialist (name and place)	☐	☐
Medical oncologist or haematologist (for adults)	☐	☐
General practitioner, or paediatrician	☐	☐
Other: (please specify)	☐	☐

Would you like to have a special follow-up programme / a special follow-up clinic based at your institution?

- ○ Yes
- ○ No

=> In your case this is the end of the questionnaire. Thank you very much for your participation!

SECTION 3: Guidelines for Long-Term Follow-Up

1. Do you use any long term follow-up guidelines? This could include published international guidelines, but also local guidelines and leaflets developed in your institution.

- ○ Yes
- ○ No   If you have answered "No" please skip question 3 to 4 on the next page.

2. Are there different guidelines for follow-up of survivors of different cancers?

- ○ Yes
- ○ No

3. Please list all the names of guidelines, sources and diagnostic groups that you use.

- If different for survivors of different cancers, please indicate the survivor group.
- If published please indicate publication.
- If not published, please briefly indicate general source and when introduced.

a) Name of guideline, source and diagnostic group:	
b) Name of guideline, source and diagnostic group:	
c) Name of guideline, source and diagnostic group:	
d) Name of guideline, source and diagnostic group:	
e) Name of guideline, source and diagnostic group:	

4. Are there survivors of certain cancers for whom NO follow-up guidelines are used?

- ○ Yes for the following diagnoses we do not use guidelines (Please indicate all diagnoses.):
- ○ No (We use guidelines for all follow-up of diagnoses.)

Thanks a lot for your participation!

If you have any further questions or comments please e-mail to: michel@ispm.unibe.ch

Sincerely yours

Institute of Social and Preventive Medicine
University of Bern
Switzerland

Reminder!

May we ask you to send us an example of an anonymised ***patient-specific treatment summary*** and the ***information about follow-up care, late effects or life after cancer*** you give patients.

E-mail to:
michel@ispm.unibe.ch

...or mail to:
Gisela Michel, PhD
Institute of Social and Preventive Medicine
University of Bern
Finkenhubelweg 11
3012 Bern
Switzerland
